# Effect of Surface Treatments and Bonding Agents on Microshear Bond Strength of Zirconia-Reinforced Lithium Silicate and Conventional Glass-Ceramics: Silane Versus Universal Adhesive

**DOI:** 10.3390/polym18151866

**Published:** 2026-07-30

**Authors:** Soner Sismanoglu, Gorkem Sengez, Aliye Tugce Gurcan, Vasfiye Isik, Zeynep Hale Keles

**Affiliations:** 1 Department of Restorative Dentistry, Faculty of Dentistry, Istanbul University-Cerrahpasa, Istanbul 34500, Türkiye; 2 Private Practice, Istanbul 34000, Türkiye; gorkemsengun@gmail.com; 3 Department of Pediatric Dentistry, Faculty of Dentistry, Dokuz Eylul University, Izmir 35380, Türkiye; aliyetugce.gurcan@deu.edu.tr; 4 Department of Endodontics, Faculty of Dentistry, Istanbul University-Cerrahpasa, Istanbul 34500, Türkiye; vasfiye86@hotmail.com; 5 Department of Restorative Dentistry, Faculty of Dentistry, Istanbul Atlas University, Istanbul 34408, Türkiye

**Keywords:** CAD/CAM ceramics, microshear bond strength, silane coupling agent, universal adhesive, surface treatment

## Abstract

This in vitro study evaluated the effects of four surface treatments (no treatment, hydrofluoric acid [HF] etching, aluminum oxide [AlO] air abrasion, and tribochemical silica coating [TSC]) and two bonding agents (dedicated silane vs. silane-containing universal adhesive) on the microshear bond strength of four CAD/CAM glass-ceramic materials, including two zirconia-reinforced lithium silicate ceramics and two conventional glass-ceramics. Four CAD/CAM materials (Celtra Duo, IPS e.max CAD, IPS Empress CAD, and Vita Suprinity) were subjected to four surface treatments (no treatment, HF, AlO air abrasion, and TSC) and further divided according to bonding agent application: dedicated silane (Clearfil Ceramic Primer Plus) or silane-containing universal adhesive (Single Bond Universal). Microshear bond strength testing was performed after 24 h of water storage (*n* = 10). Failure modes were classified under stereomicroscopy, and surface topography was evaluated by SEM. Data were analyzed using three-way ANOVA and Tukey’s HSD test (α = 0.05). All three main factors and their interactions significantly affected bond strength values (*p* < 0.05; R^2^ = 0.863). HF etching with silane produced the highest values for most materials. However, IPS Empress CAD treated with TSC and silane yielded the highest overall bond strength (25.2 MPa). Dedicated silane consistently outperformed the universal adhesive across all combinations. ZLS ceramics showed intermediate values with distinct etching patterns compared with conventional glass-ceramics. Optimal bonding protocols for CAD/CAM glass-ceramics are material-dependent. Dedicated silane application is recommended over silane-containing universal adhesives for maximum bond strength.

## 1. Introduction

The use of computer-aided design/computer-aided manufacturing (CAD/CAM) technology in restorative dentistry has expanded considerably, allowing clinicians to fabricate indirect restorations with improved precision and predictable esthetic outcomes. Currently, a wide range of CAD/CAM materials is available, including leucite-reinforced glass-ceramics, lithium disilicate glass-ceramics, zirconia-reinforced lithium silicate ceramics, and various hybrid materials, each with distinct microstructural properties and adhesive requirements [1,2]. The clinical success and longevity of these restorations depend critically on the establishment of a durable bond between the ceramic surface and the resin-based luting agent, and this bond is achieved through a combination of micromechanical retention and chemical adhesion [3].

Surface treatment of the ceramic intaglio surface is a prerequisite for achieving reliable adhesion. Etching with hydrofluoric acid (HF) is the most commonly recommended method for conditioning silica-based glass-ceramics. HF selectively dissolves the glassy matrix phase, creating a micro-retentive surface topography that facilitates the infiltration of resin-based materials [4]. The effectiveness of HF etching varies among ceramic types depending on each material’s crystalline content, glass phase composition, and microstructure [5]. Alternative surface conditioning methods include airborne particle abrasion with aluminum oxide (AlO) and tribochemical silica coating (TSC). While AlO abrasion creates surface roughness through mechanical action, TSC combines enhanced chemical reactivity with micromechanical roughening by depositing a reactive silica layer on the ceramic surface via the high-speed impact of silica-coated alumina particles [6]. Both methods have been investigated as alternatives or complements to HF etching, particularly for materials where acid application may be less effective or in situations where simplified clinical protocols are desired [7].

Following surface treatment, the application of a silane coupling agent constitutes the second key step in the adhesion protocol for silica-based ceramics. Silane molecules, most commonly γ-methacryloxypropyltrimethoxysilane (γ-MPTS), act as a chemical bridge between the inorganic ceramic surface and the organic resin matrix by forming siloxane bonds with surface hydroxyl groups and simultaneously undergoing copolymerization with methacrylate-based luting agents [8]. This combined mechanical and chemical bonding approach has been widely regarded as the standard protocol for obtaining reliable and long-lasting resin–ceramic adhesion [2].

In recent years, silane-containing universal adhesives have been introduced to the market as a simplified alternative to the traditional multi-step protocol. These products aim to eliminate the need for a separate silane application step by combining γ-MPTS or similar coupling agents with functional monomers such as 10-methacryloyloxydecyl dihydrogen phosphate (10-MDP), adhesive resins, and solvents into a single bottle [9]. However, various concerns have been raised regarding the effectiveness of the silane in these formulations. The acidic pH environment of universal adhesives (typically ranging from 2.0 to 3.0) has been shown to accelerate the hydrolysis and premature self-condensation of silane monomers, thereby reducing the number of reactive silanol groups available for bonding to the ceramic surface [10]. Additionally, the presence of Bis-GMA and other resin monomers can inhibit the silane condensation reaction, further reducing the effectiveness of the coupling mechanism [11]. Consequently, there is an ongoing debate in the literature regarding whether the silane present in universal adhesives can sufficiently replace a separate silane primer, particularly for challenging clinical applications [12,13].

Despite a significant amount of research on surface treatment methods and silane application strategies for CAD/CAM ceramics, some topics have not yet been sufficiently investigated. In particular, zirconia-reinforced lithium silicate (ZLS) ceramics represent a relatively new class of CAD/CAM materials, as their adhesive behavior has not been systematically compared with established glass-ceramics such as lithium disilicate and leucite-reinforced ceramics under the same experimental conditions. The incorporation of ZrO_2_ into the glass-ceramic matrix alters the microstructure and can influence both the response to surface conditioning and the effectiveness of silane coupling. Most existing studies have focused on a single material type or a limited number of surface treatment and bonding agent combinations, making it difficult to draw comprehensive conclusions regarding material-specific adhesive behavior. Furthermore, although comparisons between standalone silane primers and silane-containing universal adhesives are gaining increasing attention, few studies have simultaneously evaluated this comparison across multiple surface treatment methods and multiple ceramic types within a single experimental design. The interaction between material type, surface conditioning method, and bonding agent type, which is critical for evidence-based protocol selection, has not been sufficiently addressed in the literature.

Therefore, the aim of this in vitro study is to evaluate the effects of four different surface treatments (untreated control, HF etching, AlO air abrasion, and TSC) and two bonding agents (a separate silane coupling agent and a silane-containing universal adhesive) on the microshear bond strength of four CAD/CAM glass-ceramic materials (IPS Empress CAD, IPS e.max CAD, Celtra Duo, and Vita Suprinity). The null hypotheses tested were as follows: (1) the type of surface treatment will not significantly affect microshear bond strength (µSBS) values, and (2) the type of bonding agent (separate silane and silane-containing universal adhesive) will not significantly affect µSBS values.

## 2. Materials and Methods

The following four types of CAD/CAM glass-ceramic blocks were used in this in vitro study: (a) zirconia-reinforced lithium silicate ceramic (ZLS-C; Celtra Duo; Dentsply Sirona, Hanau-Wolfgang, Germany), (b) lithium disilicate glass-ceramic (LDS; IPS e.max CAD; Ivoclar Vivadent, Schaan, Liechtenstein), (c) leucite-reinforced glass-ceramic (LEU; IPS Empress CAD; Ivoclar Vivadent, Schaan, Liechtenstein), and (d) zirconia-reinforced lithium silicate glass-ceramic (ZLS-V; Vita Suprinity; VITA Zahnfabrik, Bad Säckingen, Germany). The brands, batch numbers, manufacturers, and chemical compositions of the tested materials are presented in Table 1.

### 2.1. Specimen Preparation

The CAD/CAM blocks were cut into 3 mm thick slices using a precision cutter (IsoMet High Speed Pro; Buechler, Lake Bluff, IL, USA). Specimens obtained for each CAD/CAM block were embedded in a self-cured acrylic resin (Integra; BG Dental, Ankara, Turkey) with the surfaces to be tested facing upwards. The embedded specimens were ground and finished for 30 s using water-cooled 600-grit silicon carbide (SiC) paper under constant manual pressure applied by a single operator, and then specimens were randomly assigned to one of the following surface treatment groups (*n* = 5 specimens per group):

Group 1 (Control, no treatment): no surface treatment was applied to the CAD/CAM material surface as a control.

Group 2 (HF; hydrofluoric acid etching): 4% hydrofluoric acid (Ivoclar IPS Ceramic Etching Gel; Ivoclar Vivadent, Schaan, Liechtenstein) was applied to the CAD/CAM material surfaces according to the manufacturer-recommended etching times: 20 s for LDS, 30 s for ZLS-C and ZLS-V, and 60 s for LEU. The acid was rinsed with distilled water for 30 s and then air-dried.

Group 3 (AlO; airborne particle abrasion): Airborne particle abrasion treatment was performed on CAD/CAM material surfaces using a sandblaster (Airsonic mini sandblaster; Hager Werken, Duisburg, Germany) 10 mm above the specimen surface at 2 bar pressure with AlO particles (Cobra; 50 µm AlO, Renfert GmbH, Hilzingen, Germany) for 10 s. The same application time was used for all four CAD/CAM materials. The specimens were cleaned in distilled water using an ultrasonic bath for 5 min and were then air-dried.

Group 4 (TSC; tribochemical silica coating): TSC was performed using the same sandblaster 10 mm above the specimen surface at 2 bar pressure. For TSC, silica-coated alumina particles (CoJet sand; 30 µm, 3M ESPE, Seefeld, Germany) were used for 10 s, and the same application time was used for all four CAD/CAM materials. Following TSC application, the specimens were ultrasonically cleaned in distilled water for 5 min and subsequently air-dried.

### 2.2. Microshear Bond Strength Testing

Following the surface treatments, each specimen surface was divided into two halves. A silane coupling agent (Clearfil Ceramic Primer Plus; Kuraray Noritake Dental Inc., Tokyo, Japan) was applied to one half of each specimen surface with an applicator brush in accordance with the manufacturer’s recommendations, whereas the other half of the surfaces were conditioned with a universal adhesive (SBU; Single Bond Universal; 3M ESPE, St. Paul, MN, USA) and light-cured using an LED curing unit (Elipar Deep Cure; 3M ESPE, St. Paul, MN, USA) according to the manufacturer’s recommendations. The light output was verified periodically using a radiometer. Transparent microtubules with an inner diameter of 1 mm and a height of 0.5 mm were prepared from polyvinyl tubing, and a gauge was used to ensure parallel ends. Following adhesive application, two resin microtubules were placed on each half of the specimen surface (in total, *n* = 10 for silane application and *n* = 10 for universal adhesive application in each subgroup). Each microtubule was adjusted over the treated specimen surface and gently filled with a luting resin material (Clearfil SA Cement Plus; Kuraray Noritake Dental Inc., Tokyo, Japan). This procedure resulted in small resin cylinders bonded to the treated surfaces. The luting resin was light-polymerized for 20 s using the LED curing unit according to the manufacturer’s instructions. All specimens were then stored in distilled water at 37 °C for 24 h prior to testing.

A shear force was applied to the adhesive interface using a μSBS testing device (MOD Dental, Esetron Smart Robotechnologies, Ankara, Turkey) at a crosshead speed of 0.5 mm/min. The specimen storage conditions and loading parameters were selected in accordance with the general methodological principles of ISO/TS 11405, and the microshear configuration followed protocols previously established for resin–ceramic bonding [6,14]. The load at failure was recorded in MPa. The failure modes were examined at 40× magnification with a stereomicroscope (SMZ745T; Nikon, Tokyo, Japan). Failure modes were categorized as adhesive/mixed (A/M; failure predominantly at the resin–ceramic interface), cohesive in luting cement (CLC; failure within the luting resin material), or cohesive in ceramic (CC; failure within the ceramic substrate).

### 2.3. Scanning Electron Microscopy Evaluation

For scanning electron microscopy (SEM) analysis, one representative specimen from each of the four surface treatment conditions (including the untreated control) was prepared for each of the four materials, yielding a total of 16 specimens. The specimens were sputter-coated with a thin gold layer (Polaron SC7620; ThermoVG Scientific, East Grinstead, UK) and examined under SEM (JEOL 5500; JEOL Inc., Peabody, MA, USA) at 10 kV accelerating voltage. Surface topography was assessed at 5000× magnification.

### 2.4. Statistical Analysis

For each material, surface treatment, and bonding agent combination, two resin cylinders were bonded per specimen (*n* = 5 specimens), yielding 10 bond strength measurements per experimental subgroup (*n* = 10). A total of 32 subgroups were formed by combining four materials, four surface treatments, and two bonding agents, resulting in 320 measurements overall. The mean and standard deviations were calculated, and normality of data distribution was assessed using the Shapiro–Wilk test for each group. According to the results, the data were normally distributed across all groups. Accordingly, a three-way analysis of variance (ANOVA) was used to evaluate the effects of CAD/CAM material type (four levels), surface treatment (four levels), and bonding agent (silane vs. universal adhesive), including all possible two-way and three-way interactions among these factors. Post hoc pairwise comparisons were performed using Tukey’s HSD test. The significance level was set at α = 0.05. All analyses were performed using SPSS statistical software (Version 31; IBM, Armonk, NY, USA).

## 3. Results

### 3.1. Microshear Bond Strength Analysis

A three-way ANOVA revealed that all three main factors (material type, surface treatment, and bonding agent [silane and universal adhesive]) had statistically significant effects on µSBS values (*p* < 0.001 for all; Table 2). All two-way interactions (material × surface treatment, material × bonding agent, surface treatment × bonding agent) and the three-way interaction (material × surface treatment × bonding agent) were also found to be significant (*p* < 0.05), indicating that the effect of each factor depends on the levels of the other two. The model explained 86.3% of the total variance (R^2^ = 0.863, adjusted R^2^ = 0.848).

The mean and standard deviation values of µSBS for each experimental group are presented in Table 3. For all materials, the control groups (without surface treatment) yielded the lowest bond strength values, ranging from 5.2 ± 1.9 to 10.1 ± 1.6 MPa. Etching with hydrofluoric acid produced the highest µSBS values, ranging from 16.6 ± 3.0 to 22.3 ± 2.3 MPa, for most material and bonding agent combinations. AlO air abrasion and TSC yielded intermediate values.

Among the materials tested, the combination of HF etching and silane application on LDS produced one of the highest µSBS values (22.3 ± 2.3 MPa). However, the highest overall bond strength was observed in LEU treated with a combination of TSC and silane application (25.2 ± 1.4 MPa); this value was significantly higher than that of all other groups of the same material and clearly exceeded the corresponding HF group (20.8 ± 1.1 MPa). This pattern is specific to LEU, as in the remaining three materials, acid etching with HF consistently yielded the highest or among the highest µSBS values.

Silane application generally yielded higher µSBS values compared to the universal adhesive (Single Bond Universal) in all material and surface treatment combinations. The difference between silane and SBU was found to be statistically significant in four groups: LDS control (*p* = 0.0005), LDS HF (*p* < 0.0001), LEU TSC (*p* < 0.0001), and ZLS-V TSC (*p* = 0.0004). Although silane consistently produced numerically higher values in the remaining groups, the differences did not reach statistical significance.

LDS demonstrated a distinct response to the bonding agent in the control group: the control value for the silane-treated group (10.1 ± 1.6 MPa) was nearly twice that of the SBU-treated control group (5.2 ± 1.9 MPa; *p* = 0.0005). This significant difference was not observed in the other three materials, and their bond strengths remained comparable to those of the silane and SBU groups (ZLS-C: 7.6 and 7.1; LEU: 7.2 and 6.8; ZLS-V: 8.1 and 6.6; all *p* > 0.05).

### 3.2. Failure Mode Analysis

The failure mode distributions are presented in Table 4. Adhesive/mixed failures were the predominant failure type in all groups. Cohesive failure (CC) within the ceramic substrate was not observed in any group. Cohesive failures (CLC) within the luting cement were observed only in the groups that underwent surface treatment, and their frequency tended to increase with higher µSBS values. The highest CLC failure rate was observed in the LDS HF group with silane (4/10) and in the LEU groups treated with silane, HF, and TSC (3/10 each). All control groups exhibited only adhesive/mixed failure (10/10) regardless of the bonding agent, a finding consistent with the low bond strength values observed in these groups.

### 3.3. SEM Evaluation

**Figure 1:**
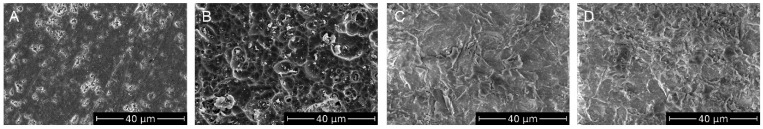
Representative SEM micrographs (5000×) of Celtra Duo (ZLS-C) surfaces: (**A**) control (no treatment), (**B**) hydrofluoric acid etching, (**C**) aluminum oxide air abrasion, and (**D**) tribochemical silica coating.

**Figure 2:**
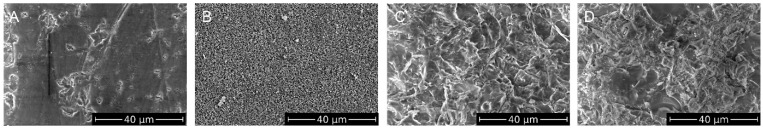
Representative SEM micrographs (5000×) of IPS e.max CAD (LDS) surfaces: (**A**) control (no treatment), (**B**) hydrofluoric acid etching, (**C**) aluminum oxide air abrasion, and (**D**) tribochemical silica coating.

**Figure 3:**
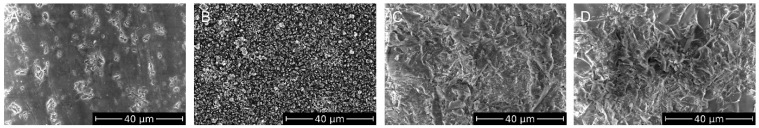
Representative SEM micrographs (5000×) of IPS Empress CAD (LEU) surfaces: (**A**) control (no treatment), (**B**) hydrofluoric acid etching, (**C**) aluminum oxide air abrasion, and (**D**) tribochemical silica coating.

**Figure 4:**
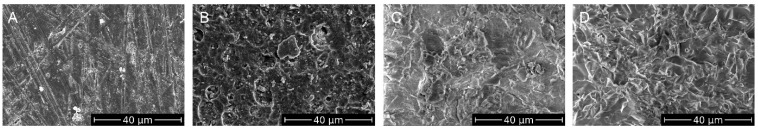
Representative SEM micrographs (5000×) of Vita Suprinity (ZLS-V) surfaces: (**A**) control (no treatment), (**B**) hydrofluoric acid etching, (**C**) aluminum oxide air abrasion, and (**D**) tribochemical silica coating.

Representative SEM micrographs of treated ceramic surfaces at 5000× magnification are presented in Figure 1, Figure 2, Figure 3 and Figure 4. Control samples (Panel A) exhibited relatively smooth surfaces containing minor irregularities resulting from the polishing process. HF-etched surfaces (Panel B) exhibited characteristic micro-retentive patterns featuring micro-pores and increased surface area due to significant surface dissolution. The etching pattern varied among materials: LDS exhibited a homogeneous, fine-grained porous structure, while LEU and ZLS-V exhibited a coarser etching pattern. Surfaces treated with AlO air abrasion (Panel C) exhibited irregular, rough topographies with sharp-edged features and evidence of surface fracturing. Surfaces treated with a TSC (Panel D) showed surface roughening similar to the AlO-treated groups but exhibited a slightly less aggressive surface alteration pattern.

## 4. Discussion

This study evaluated the effects of different surface treatments and the application of a silane-containing universal adhesive with a separate silane coupling agent on the microshear bond strength values of four CAD/CAM glass-ceramic materials. The findings revealed that all three main factors (material type, surface treatment, and bonding agent), as well as their interactions, significantly influenced the µSBS values. These results indicate that the bonding behavior of CAD/CAM ceramics cannot be predicted by a single variable but rather depends on the specific combination of the material used, the surface conditioning method, and the coupling strategy. This material-dependent response is consistent with the findings of previous studies investigating adhesive protocols for CAD/CAM restorations [1,14]. The evaluation of two ZLS ceramics (ZLS-C and ZLS-V) alongside two established glass-ceramics (LDS and LEU) within the same experimental design allowed for a direct comparison of these new zirconia-containing formulations with traditional materials. ZLS ceramics generally produced intermediate levels of bond strength; SEM observations revealed a granular, spheroidal etching pattern following HF etching, which differed from the classic honeycomb-like microporosity observed in LDS and LEU; this reflects the effect of ZrO_2_ on the dissolution behavior of the glass matrix. A previous study on ZLS ceramics exposed to different etching protocols reported similar morphological findings and confirmed that the presence of zirconia in the glass-ceramic matrix alters the surface response to HF conditioning [15]. Based on these findings, both null hypotheses were rejected: the type of surface treatment significantly affected µSBS values (*p* < 0.001), and the type of bonding agent significantly affected bond strength in all materials (*p* < 0.001).

Across all materials and surface treatments, the application of a separate silane coupling agent yielded numerically higher µSBS values compared to Single Bond Universal, a silane-containing universal adhesive. Although this difference reached statistical significance in only four of the sixteen material–surface treatment combinations, the consistency of the pattern across all 32 comparisons is noteworthy. Similar trends have been reported in studies comparing separate silane primers with silane-containing universal adhesives on various glass-ceramic substrates [8,12,16]. A recent investigation evaluating the microshear bond strength of four silane-containing universal adhesives to lithium disilicate also confirmed that a separate silane application significantly improved bond strength for most adhesive systems tested [17]. The superiority of the separate silane application can be attributed to several factors. In universal adhesives, the silane monomer is present in an acidic aqueous environment (pH 2.7 for SBU), and it has been demonstrated that this accelerates the hydrolysis and early self-condensation of γ-MPTS, thereby reducing the number of reactive silanol groups available for bonding to the ceramic surface [10]. In addition, Bis-GMA present in the universal adhesive can inhibit the condensation reaction between the ceramic’s hydroxyl groups and the silane’s silanol groups via a mechanism explained by Le Chatelier’s principle; in this mechanism, excess resin components prevent effective water removal during condensation [11]. The relatively high viscosity of universal adhesives can weaken micromechanical locking by further limiting their ability to penetrate the micro-retentive features created by surface treatments [1]. In a comprehensive study evaluating five different silane strategies on five CAD/CAM materials, the traditional three-step silane protocol consistently outperformed silane-containing universal adhesives by approximately 2 to 3 MPa, regardless of the ceramic type [2]. Current findings are consistent with this observation and support the recommendation that a separate silane application step should not be omitted when optimal bond strength is desired. However, it should also be noted that the universal adhesive achieved clinically acceptable bond strength values in all groups that underwent surface treatment; this is consistent with studies supporting its use as a practical alternative in clinical settings where procedural efficiency is prioritized [9,18,19]. Studies evaluating the performance of universal adhesives on leucite-reinforced ceramics have also reported acceptable but generally lower bond strength values compared to separate silane primers [12,20]. These consistent findings further support the rejection of the second null hypothesis, indicating that the type of bonding agent influences the µSBS value regardless of the combination of material and surface treatment tested.

The significant effect of surface treatment on µSBS was an expected result, as all three conditioning methods (HF etching, AlO air abrasion, and TSC) are well-established approaches for strengthening the resin–ceramic bond [3]. Etching with hydrofluoric acid generally produced the highest bond strength values across all four tested materials. This finding is consistent with the understanding that HF selectively dissolves the glassy matrix of silicate-based ceramics, creating a micro-retentive surface topography that facilitates resin infiltration and micromechanical interlocking [4,21]. It is known that the effect of HF etching varies depending on the ceramic composition and the ratio of crystalline to glassy phases, with each material exhibiting a unique etching pattern [4]. A systematic review and meta-analysis evaluating the effects of HF concentration on the bond strength of glass-ceramics confirmed that etching time and ceramic type are critical variables affecting bond performance [5]. A study on ZLS ceramics also demonstrated that the bond strength between resin cement and ZLS is significantly influenced by the type of surface treatment, with HF etching followed by silanization yielding favorable immediate results [22]. A comparative investigation on CAD/CAM glass-ceramics further confirmed that HF acid etching can be recommended for feldspathic, lithium disilicate, and zirconia-reinforced ceramics, yielding higher bond strengths than sandblasting and silica coating [23]. The SEM observations in this study support these findings, as the HF-etched surfaces exhibited distinct microporous patterns that differed morphologically from the other tested materials. AlO air abrasion and TSC produced intermediate values in most material groups, creating surface roughness through mechanical action rather than chemical dissolution. It is known that these mechanical methods increase surface area and wettability, while also leaving residual alumina or silica particles that can enhance chemical bonding following silane application [6].

A notable finding of this study is the high µSBS value (25.2 ± 1.4 MPa) observed in the LEU group treated with TSC and silane application; this value exceeded that of both the corresponding AlO group and the HF-etched group of the same material. This behavior is specific to LEU and was not observed in the other three materials, where HF etching consistently yielded the highest or among the highest values. The TSC process deposits a thin silica layer onto the ceramic surface through the high-speed impact of silica-coated alumina particles, creating a chemically reactive, silica-rich surface suitable for both micromechanical roughness and silane coupling. The leucite crystalline phase in LEU, which constitutes a substantial proportion of the material volume, may provide a particularly suitable substrate for this mechanism. Leucite crystals are inherently rich in silica and potassium aluminosilicate, and the tribochemical process may increase the availability of silica on the surface beyond what is achieved through selective glass phase dissolution by HF etching. This finding is supported by previous reports indicating that TSC is a particularly effective conditioning method for leucite-reinforced ceramics [7]. In a study evaluating surface treatments for bonding orthodontic brackets to silicate-based ceramics, the combination of TSC and silanization produced the highest bond strength values, surpassing those obtained by HF etching [24]. Additionally, a study investigating repair protocols for different types of glass-ceramics reported that TSC is applicable to leucite-reinforced ceramics and may represent an effective alternative to HF etching for this specific material type [7]. Interestingly, in a study comparing surface conditioning protocols for ZLS and lithium disilicate ceramics, the CoJet (tribochemical) group produced the highest repair bond strength for both LDS and ZLS-V, exceeding the values obtained with HF etching alone [25]. This finding from a different material group further supports the potential of TSC as an effective conditioning method, particularly when combined with dedicated silanization. The low standard deviation (1.4 MPa) observed in the LEU TSC+silane group, when compared to other high-performance groups, suggests a more predictable and consistent bonding mechanism at this specific material–surface treatment interface. A previous study on the surface treatment of similar silicate-based CAD/CAM materials also reported positive results obtained with TSC, particularly when combined with a separate silane application [6,26].

The LDS material exhibited a unique response in the control group that was dependent on the bonding agent; the control value for the silane-treated group (10.1 ± 1.6 MPa) was nearly twice that of the SBU-treated control group (5.2 ± 1.9 MPa). This significant difference was not observed in the other three materials. The lithium disilicate microstructure of LDS, characterized by interlocking needle-like crystals and offering limited exposure of the glassy matrix on untreated surfaces, may provide fewer accessible silanol sites for effective interaction with the silane component in the universal adhesive. When applied in a carrier solution with lower viscosity and at a more suitable pH, the separate silane appears more capable of wetting and reacting with the limited reactive regions on the unmodified LDS surface. This observation is consistent with reports suggesting that LDS requires more aggressive surface conditioning for reliable bonding and that the silane in universal adhesives may be insufficient in cases where micromechanical retention is minimal [11,25,27]. A study evaluating the effect of silane-containing universal bonding agents on lithium disilicate also demonstrated that the application of a separate silane treatment resulted in significantly higher bond strengths compared to the use of universal adhesives alone [9]. Another study investigating the shear bond strength of lithium disilicate using different surface treatments confirmed that silane application following HF etching significantly enhanced bond performance compared to HF etching alone [28]. A comparison between HF and a single-component self-etching primer on LDS and ZLS-V further demonstrated that the adhesive performance is material-specific and that simplified conditioning approaches may not be equally effective across different glass-ceramic compositions [29]. Interestingly, when surface treatment was applied, the difference between silane and SBU for LDS narrowed in the mechanical treatment groups (AlO and TSC), suggesting that the formation of sufficient micromechanical retention partially compensates for the limitations of silane within the universal adhesive. The long-term clinical implications of this observation require further research; a previous study demonstrated that a silane and adhesive combination containing MDP provided superior bond strength to lithium disilicate over a 1-year period compared to adhesive application alone [8], and recent evidence suggests that even clinical procedures such as try-in may compromise bond stability to lithium disilicate, further emphasizing the sensitivity of the resin–ceramic interface [30]. Furthermore, the effects of surface treatment and thermocycling on ZLS ceramics showed that while most surface-treated groups were significantly affected by thermal aging, the sandblasting and silane group maintained stable bond strength values, highlighting the importance of treatment selection for long-term performance [31]. A study evaluating surface treatments of ZLS ceramic bonded to resin cement also reported that thermocycling promoted different levels of adhesive strength and that failures were predominantly adhesive in nature [32].

Failure mode analysis revealed a pattern consistent with the µSBS data. All control groups exhibited only adhesive/mixed failure, reflecting the weak interfacial bond in the absence of surface treatment. The proportion of cohesive failure within the luting cement increased with higher bond strength values, particularly in the HF and TSC groups combined with silane application. The complete absence of cohesive failure within the ceramic substrate in all groups indicates that the cohesive strength of the tested ceramics consistently exceeded the adhesive bond strength at the resin–ceramic interface, a characteristic observation in µSBS studies of CAD/CAM materials [2,14]. A systematic review evaluating the application of adhesive layers on glass-ceramics has also reported a similar relationship between bond strength values and failure mode distributions [3].

Several limitations of this study should be acknowledged. While the in vitro design provided standardized experimental conditions, it cannot fully simulate the complexity of the oral environment, which involves occlusal loading, pH variations, enzymatic activity, and biofilm formation. The specimens were soaked in distilled water for only 24 h without thermocycling, which limits the assessment of long-term bond strength. Previous studies have shown that thermal cycling significantly reduces bond strength in all bonding protocols, but the relative order of surface treatments and bonding strategies tends to be preserved after aging [2,10]. A recent systematic review also confirmed that simplified bonding strategies exhibit greater susceptibility to aging compared to traditional multi-step protocols [12]. Future studies incorporating artificial aging via thermocycling and prolonged water immersion will provide clinically relevant information regarding bond stability. Although manufacturer-recommended etching times were followed in this study, the optimal HF etching duration for each ceramic type remains a subject of investigation, as different ceramics may exhibit varying responses to different HF exposure times and concentrations [4,5]. The use of a single luting agent and a single universal adhesive also limits the generalizability of the findings to other product combinations. In addition, failure modes were classified under stereomicroscopy at the time of testing, but representative photomicrographs were not archived; future studies should document failure patterns photographically to complement the quantitative distribution. Despite these limitations, the significant three-way interaction observed in the ANOVA highlights the complexity of the resin–ceramic bond and emphasizes the importance of considering specific material, surface treatment, and bonding agent combinations rather than applying a universal protocol.

## 5. Conclusions

Within the limitations of this in vitro study, the combination of etching with hydrofluoric acid and a separate silane application yielded the highest bond strength values for most of the tested materials. However, the combination of TSC and silane produced the highest overall bond strength for LEU, indicating a material-specific advantage of this surface conditioning method for leucite-reinforced glass-ceramics. While the separate silane application consistently outperformed the silane-containing universal adhesive across all material and surface treatment combinations, this difference was found to be statistically significant only in a limited number of groups. These findings indicate that optimal adhesive protocols for CAD/CAM glass-ceramics must be tailored to the specific material type and that a separate silane application step continues to be recommended when maximum bond strength is desired.

## Figures and Tables

**Table 1:** Materials used in the study.

Material	Batch Number	Type	Composition
Celtra Duo (ZLS-C; Dentsply Sirona Inc., Hanau-Wolfgang, Germany)	18027273	Zirconia-reinforced lithium silicate glass-ceramic	Li_2_Si_2_O_5_ Filler: 58% SiO_2_, 18.5% Li_2_O, 5% P_2_O_5_, 1.9% Al_2_O_3_, 1% Tb_2_O_3_, and 2% CeO_2_, 10.1% ZrO_2_ dissolved in the glass matrix by weight
IPS e.max CAD (LDS; Ivoclar Vivadent, Schaan, Liechtenstein)	W42353	Lithium disilicate glass-ceramic	Li_2_Si_2_O_5_ Filler: 57–80% SiO_2_, 11–19% Li_2_O, 0–13% K_2_O, 0–11% P_2_O_5_, 0–8% ZrO_2_, 0–8% ZnO, 0–5% Al_2_O_3_, 0–5% MgO, 0–8% pigment by weight
IPS Empress CAD (LEU; Ivoclar Vivadent, Schaan, Liechtenstein)	X23271	Leucite-reinforced glass-ceramic	KAlSi_2_O_6_ Filler: 60–65% SiO_2_, 16–20% Al_2_O_3_, 10–14% K_2_O, 3.5–6.5% Na_2_O, 0.7–8% CaO, and oxide pigments by weight
Vita Suprinity (ZLS-V; VITA Zahnfabrik, Bad Säckingen, Germany)	63200	Zirconia-reinforced lithium silicate glass-ceramic	Li_2_Si_2_O_5_ Filler: 56–64% SiO_2_, 15–21% Li_2_O, 8–12% ZrO_2_, 3–8% P_2_O_5_, 1–4% K_2_O, 0–4% CeO_2_, 1–4% Al_2_O_3_, 0.1% La_2_O_3_, 0–6% pigment by weight

Abbreviations: lithium disilicate (Li_2_Si_2_O_5_), potassium aluminum silicate (leucite crystals; (KAlSi_2_O_6_), silicon dioxide (SiO_2_), lithium oxide (Li_2_O), diphosphorus pentoxide (P_2_O_5_), aluminum oxide (Al_2_O_3_), terbium oxide (Tb_2_O_3_), cerium dioxide (CeO_2_), zirconium dioxide (ZrO_2_), potassium oxide (K_2_O), zinc oxide (ZnO), magnesium oxide (MgO), sodium oxide (Na_2_O), calcium oxide (CaO), and lanthanum oxide (La_2_O_3_).

**Table 2:** Three-way ANOVA results for the effects of material type, surface treatment, and bonding agent on µSBS values.

Source	Type III Sum of Squares	Df	Mean Square	F	Sig.
Corrected Model	8609.035 ^a^	31	277.711	58.437	0.000
Intercept	67,529.631	1	67,529.631	14,209.971	0.000
Material	304.405	3	101.468	21.352	0.000
Treatment	6900.504	3	2300.168	484.015	0.000
Silane	572.985	1	572.985	120.571	0.000
material × treatment	546.313	9	60.701	12.773	0.000
material × silane	44.702	3	14.901	3.135	0.026
treatment × silane	86.885	3	28.962	6.094	0.000
material × treatment × silane	153.240	9	17.027	3.583	0.000
Error	1368.654	288	4.752		
Total	77,507.320	320			
Corrected Total	9977.689	319			

^a^ R^2^ = 0.863 (adjusted R^2^ = 0.848). The “Silane” factor refers to the bonding agent variable (dedicated silane vs. silane-containing universal adhesive).

**Table 3:** Microshear bond strength values (MPa) for each material, surface treatment, and bonding agent combination.

		Mean (SD)		
Material	Surface Treatment	With Silane	With SBU	*p* Value
Celtra Duo	Control	7.6 (2.0) ^D^	7.1 (1.4) ^D^	>0.999
	HF	21.2 (2.0) ^BC^	19.5 (2.1) ^A^	>0.999
	AlO	14.8 (3.0) ^E^	12.1 (2.4) ^C^	0.958
	TSC	16.1 (2.6) ^DE^	12.9 (3.9) ^C^	0.507
IPS e.max	Control	10.1 (1.6) ^F^	5.2 (1.9) ^D^	0.0005
	HF	22.3 (2.3) ^AB^	16.6 (3.0) ^AB^	<0.0001
	AlO	14.2 (1.7) ^E^	12.1 (1.8) ^C^	>0.999
	TSC	16.0 (1.3) ^DE^	12.9 (2.1) ^C^	0.556
Empress	Control	7.2 (1.2) ^F^	6.8 (1.4) ^D^	>0.999
	HF	20.8 (1.1) ^BC^	19.9 (1.9) ^A^	>0.999
	AlO	16.2 (1.5) ^DE^	14.6 (2.0) ^BC^	>0.999
	TSC	25.2 (1.4) ^A^	18.6 (2.1) ^A^	<0.0001
Vita Suprinity	Control	8.1 (1.2) ^F^	6.6 (1.8) ^D^	>0.999
	HF	20.3 (1.5) ^BC^	19.6 (1.4) ^A^	>0.999
	AlO	16.1 (3.5) ^DE^	13.3 (2.6) ^BC^	0.922
	TSC	18.0 (3.4) ^CD^	13.1 (2.9) ^BC^	0.0004

Values are presented as mean (standard deviation) in MPa. Superscript letters indicate Tukey’s HSD post hoc groupings within each bonding agent column; groups sharing the same letter are not significantly different across all material and surface treatment combinations (α = 0.05). The *p* value column represents the comparison between silane and SBU for the same material and surface treatment combination. SBU, Single Bond Universal; HF, hydrofluoric acid; AlO, aluminum oxide; TSC, tribochemical silica coating.

**Table 4:** Failure mode distribution according to material type, surface treatment, and bonding agent (*n* = 10).

		Fracture Mode					
		With Silane			With SBU		
Material	Surface Treatment	A/M	CLC	CC	A/M	CLC	CC
Celtra Duo	Control	10	0	0	10	0	0
	HF	8	2	0	8	2	0
	AlO	10	0	0	9	1	0
	TSC	9	1	0	9	1	0
IPS e.max	Control	10	0	0	10	0	0
	HF	6	4	0	8	2	0
	AlO	8	2	0	10	0	0
	TSC	8	2	0	9	1	0
Empress	Control	10	0	0	10	0	0
	HF	7	3	0	8	2	0
	AlO	9	1	0	9	1	0
	TSC	7	3	0	8	2	0
Vita Suprinity	Control	10	0	0	10	0	0
	HF	8	2	0	8	2	0
	AlO	9	1	0	9	1	0
	TSC	8	2	0	9	1	0

Values represent the number of specimens exhibiting each failure mode. A/M, adhesive/mixed failure at the resin–ceramic interface; CLC, cohesive failure within the luting cement; CC, cohesive failure within the ceramic substrate. SBU, Single Bond Universal; HF, hydrofluoric acid; AlO, aluminum oxide; TSC, tribochemical silica coating.

## Data Availability

The original contributions presented in this study are included in the article. Further inquiries can be directed to the corresponding authors.
